# Exploring the Influence of Parity on Metabolic Profile, Performance and Offspring Growth in *Bos indicus* Beef Cows

**DOI:** 10.3390/vetsci12121215

**Published:** 2025-12-18

**Authors:** Isabela I. Rodrigues, Matheus L. Ferreira, Luciana N. Rennó, Naiara A. Marcos, Ronaldo G. da Silva Júnior, Isabelle P. Siqueira, Camila de P. Magalhães, Edenio Detmann, Sebastião de C. Valadares Filho

**Affiliations:** 1 Departament of Animal Science, Universidade Federal de Viçosa, Viçosa 36570, MG, Brazil; 2 Hill Farm Research Station, Louisiana State University Agricultural Center, Homer, LA 71040, USA; 3 Campus Alfenas, Universidade Professor Edson Antônio Velano-Unifenas, Alfenas 37132, MG, Brazil

**Keywords:** blood biochemical indicators, grazing, physiology, peripartum

## Abstract

The objective of this study was to evaluate how parity (primiparous vs. multiparous) influences metabolic profile, performance, and offspring growth in grazing Nellore cows, and to explore potential associations among key metabolic traits, milk yield, and performance indicators. Thirty-four pregnant Nellore cows at approximately 230 ± 20 days of gestation were sorted by parity group and randomly allocated into one of six *Uruchloa decumbens* pastures, totaling 17 multiparous cows (597.8 ± 39 kg; age = 4 to 6 yrs) and 17 primiparous cows (407 ± 33 kg, age = 2 to 3 yrs). Considering calving day as day 0, cow full BW and BCS were collected on days −63, −21, −7, 0, 7, 21, 63, 91, 140 and 203. Cow blood samples were collected on days −21, −14, −7, 0, 7, 14, 21, 42, 63, 91, 140 and 203. Milk yield was measured in the following day after all blood collections. Primiparous cows had higher glucose on days −7 and 0 (*p* = 0.04), greater NEFA concentration from −14 to 63 days (*p* < 0.001), and lower IGF-1 concentration from days 7 to 42 but higher on days 140 and 203 (*p* < 0.001). They also exhibited lower total proteins, albumin, and globulins, but higher serum urea N concentration (*p* < 0.001). Primiparous cows also had lower milk yield (*p* = 0.02), calf birth (*p* = 0.02) and weaning weights (*p* = 0.01) compared with multiparous cows. Pearson correlation analysis revealed distinct metabolic and physiological interrelationships across gestational and lactational stages in multiparous and primiparous cows. Overall, multiparous cows showed a less catabolic metabolic profile during the periparturient period to lactation. Primiparous cows experienced a more intense mobilization of body reserves during the early lactation period, but a more positive nutritional status towards late lactation, evidenced by recovery of IGF-1 levels and body weight gain.

## Simple Summary

This study evaluated how parity affects metabolism, performance, and calf growth in grazing Nellore cows. Thirty-four pregnant cows were monitored from late gestation to late lactation for body weight, body condition, blood metabolites, and milk yield. Primiparous cows showed higher indicators of fat mobilization (NEFA) and lower IGF-1 during early lactation, and produced less milk, resulting in lighter calves at birth and weaning. Multiparous cows maintained a more balanced metabolic profile. These findings highlight the importance of targeted nutritional strategies for primiparous cows to improve productivity in pasture-based beef systems.

## 1. Introduction

Breeding heifers early so they calve for the first time at approximately 24 months of age is essential to maximize lifetime productivity and improve economic returns for beef producers [1]. However, this means that heifers encounter the physiological demands of reproduction and lactation at an earlier stage of maturity, making adaptation during the periparturient period (approximately 3 weeks before calving through 3 weeks after calving) particularly critical [2]. This transition phase is characterized by increases in nutrient and energy demands, and hormonal and metabolic adjustments [3]. In this context, primiparous cows face unique challenges compared to multiparous cows because they must allocate nutrients not only for fetal development and milk synthesis but also for their own growth [3]. These competing demands often result in altered metabolic and hormonal profiles, which can influence performance outcomes such as body condition score (BCS), milk yield [4,5] and reproductive efficiency [6]. Blood concentrations of glucose, non-esterified fatty acids (NEFA), insulin-like growth factor 1 (IGF-1), and other blood metabolites can negatively impact animal productivity if altered. Glucose is the primary energy source for fetus and milk synthesis and NEFA reflects the mobilization of body fat reserves under negative energy balance. IGF-1 plays a key role in regulating growth, development, and metabolic processes [7], serving as a marker of nutritional status and growth potential. Low IGF-1 concentrations generally indicate poor nutritional status or energy deficiency, whereas higher concentrations are associated with better growth potential and improved nutritional status [7].

Research has extensively examined how different nutritional strategies influence the physiology of gestation and lactation in beef cows [8,9], but specific effects of parity have received comparatively less attention. Studies with dairy cows [5,10,11] have demonstrated that metabolism can be impaired by parity groups; however, information for beef cows, particularly in grazing systems, remains limited [4]. Additionally, some commonly reported physiological parameters, such as IGF-1 and NEFA, vary inconsistently among different parity groups [5,10]. These metabolic indicators provide useful cues as to the underlying processes that reflect the nutritional status of the animal [8]. Moreover, the study of metabolic and hormonal profiles has proven valuable in characterizing critical periods such as late gestation and early lactation.

While correlations between metabolites, hormones, and performance outcomes have been established in dairy cows [5,10], to the best of our knowledge, no studies have examined such associations in beef cows (i.e., body weight, BCS, and milk production). In this context, beyond exploring the changes in physiological responses across parity order, it is also important to investigate the relationship between performance indicators and metabolic profile. Such knowledge would help identify the most suitable parameters to illustrate changes in metabolism during critical physiological stages.

We hypothesize that the parity group influences metabolism in grazing Nellore cows, with primiparous cows expected to exhibit more unstable metabolic characteristics and lower milk production compared to multiparous cows. The objective of this study was to evaluate the influence of parity (primiparous vs. multiparous) on the metabolic profile, performance, and offspring growth of grazing Nellore cows, and to determine the relationships between key metabolic traits, cow performance, and milk production. Parity in this study inherently includes differences in age, body weight, and physiological maturity, thus reflecting a broader physiological condition.

## 2. Materials and Methods

The experiment was conducted at the UEPE-GC Department of Animal Science, Uni-versidade Federal de Viçosa, Viçosa, Minas Gerais, Brazil, from August 2020 to June 2021. All animal care and handling procedures were approved by the Animal Care and Use Committee of the Universidade Federal de Viçosa, protocol CEUAP-UFV 42/2020 (approval date: 18 August 2020).

### 2.1. Experimental Design and Animal Management

Thirty-four pregnant Nellore cows at approximately 230 ± 20 days of gestation were used in the study. Cows were sorted by parity group and randomly allocated into 1 of 6 *Uruchloa decumbens* pastures, totaling 17 multiparous cows (597.8 ± 39 kg; age = 4 to 6 yrs) and 17 primiparous cows (407 ± 33 kg, age = 2 to 3 yrs), with 2 or 3 cows from each parity per pasture (total of 5 or 6 cows per pasture/7 to 8 ha pastures). Cows used in the present study were confirmed pregnant by fixed-time artificial insemination in the previous breeding season using one Red Angus sire. Sample size was calculated based on previous study by Ferreira et al. [4].

All cows received an energy-protein supplement composed of corn meal (41.2%), soybean meal (56.3%), and a urea–ammonium sulfate mixture (2.5%), formulated to provide 35% crude protein (CP). The supplement was offered at a rate of 1 kg per animal per day, starting approximately 60 days before the expected calving date. Mineral mix was provided ad libitum in additional feeders [calcium phosphate (50.0%), sodium chloride (47.8%), zinc sulfate (1.4%), copper sulfate (0.70%), cobalt sulfate (0.05%), potassium iodate (0.05%), and manganese sulfate (0.025%)]. During calving season, cows were monitored every two hours for signs of calving, and calves were weighed and tagged. After calving, cow–calf pairs remained in the same pasture until the end of the study. During the breeding season, approximately 60 days after calving, cows were subjected to a fixed-time artificial insemination using a 7-day CO-Synch + CIDR protocol.

Calves were offered 5 g/kg of body weight (BW) of an energy-protein supplement formulated to contain 20% CP in a creep-feeding system from 90 days of age until weaning (d203, end of the study).

### 2.2. Sample Collection

#### 2.2.1. Cow Performance and Physiology

Considering calving day as day 0, cow full BW was collected on days −63, −21, −7, 0, 7, 21, 63, 91, 140 and 203, and BCS was collected on d−63, −21, 0, 21, 63, 91, 140 and 203. Cow blood samples were collected on days −21, −14, −7, 0, 7, 14, 21, 42, 63, 91, 140 and 203 via jugular vein using commercial vacuum tubes containing EDTA fluoride for plasma harvest and tubes containing no additives for serum harvest (BD Vacutainer, 10 mL; Becton, Dickinson and Company, São Paulo, Brazil). Pre-calving sampling was scheduled based on the expected calving date, calculated using an average gestation length of approximately 295 days for Nellore cows. Samples were initially collected at predetermined time points (e.g., −21 and −7 days before the expected calving date). When calving did not occur as predicted, sampling continued at 7-day intervals until parturition.

Blood samples were placed on ice immediately following collection and centrifuged at 2200× *g* for 20 min at 4 °C for serum and plasma harvest. Serum and plasma samples were frozen at −20 °C and stored until further analysis. Serum samples were analyzed for concentrations of albumin, total protein, urea N, creatinine, total cholesterol, high-density lipoprotein (HDL), low-density lipoprotein (LDL), NEFA, β-hydroxybutyrate (βHB) and IGF-1. Plasma samples were analyzed for concentrations of glucose.

Milking collections were performed in the morning and afternoon on the day following blood sample collection on days 8, 15, 22, 43, 64, 92, 141 and 204 to measure milk yield. Milking procedures were performed using an electric milking machine as described by Ferreira et al. [12] with a controlled suckling period before the calf separation. Briefly, to empty udders, calves were removed from their dams at 3:00 pm and were returned at 5:45 pm for a brief suckling period until 6:00 pm, after which they were separated again. The first milking occurred the following morning at 6:00 am. Prior to milking, each cow received an intravenous injection of 10 IU oxytocin (10 IU/mL; Ocitovet, Nova Alvorada, Brazil) into the mammary vein to facilitate milk letdown. Milk yield was recorded, and the exact completion time for each cow was noted. After the morning milking, cows remained separated from their calves until the afternoon milking at 6:00 pm. Total milk yield was calculated as the sum of both morning and afternoon milking. Additionally, 30 mL of milk from each cow was sampled for milk composition analyses.

#### 2.2.2. Forage Sampling

Every 30 d, pastures were sampled to determine forage mass and nutritive value. Hand-plucked samples were used to evaluate forage nutritive value. For forage mass, samples were collected by cutting at the ground level from five delimited areas (0.5 × 0.5 m), selected randomly in each paddock to quantify forage mass. Forage samples collected for nutritive value were dried in a forced-air oven (55 °C) for 72 h, ground at 1-mm and 2-mm sieve in a Wiley mill (model 3, Arthur H. Thomas, Philadelphia, PA, USA).

All data from each month were combined and expressed as seasonal averages, as follows: dry season, July and August (beginning of the experiment); dry-to-rainy transition season, September to November; rainy season, December to February; and rainy-to-dry transition season, March to May (end of the experiment). The average forage dry matter (DM) availability was: dry season = 2.6 t/ha; dry-to-rainy transition = 3.51 t/ha; rainy season = 3.18 t/ha; rainy-to-dry transition = 3.06 t/ha. The chemical composition of the forage and supplement according to season are presented in Table 1.

### 2.3. Laboratory Analysis

Blood concentrations of urea (K056), total protein (K031), albumin (K031), triglycerides (K117), total cholesterol (K083), HDL (K071), and glucose (K082) were determined using Bioclin^®^ kits (Bioclin, Belo Horizonte, Brazil). Non-esterified fatty acids and βHB were analyzed using Randox^®^ kits (Randox, FA115 and RB1007, Antrim, UK). All the abovementioned analyses were determined by the chemiluminescence method in an automated biochemical analyzer (BS200E, Mindray, Shenzhen, China) as per manufacturer instructions. Insulin-like growth factor 1 contents were quantified using Siemens^®^ kits (Siemens, Berlin, Germany) in an automated chemiluminescence analyzer (Siemens, Berlin, Germany).

LDL was estimated using the equation: TC = HDL + LDL + VLDL, where TC = total cholesterol and VLDL = triglycerides ÷ 5 [13]. Globulins were calculated by the difference between total proteins and albumin [14]. Serum urea nitrogen (SUN) was estimated to be 46.67% of total serum urea.

Milk samples were analyzed for protein, fat, lactose, and total solids contents using infrared spectroscopy (Milko Scan FT120, Foss, São Paulo, Brazil).

### 2.4. Statistical Analysis

Data was analyzed based on the following statistical model:

Y_ijk_ = μ + P_i_ + C_j_ + e_(ij)k_

where Y_ijk_ = observation taken of animal k, pertaining to parity j, within paddock i; μ = overall constant; P_i_ = paddock effect I (random); C_j_ = category (parity) effect j (fixed) e_(ij)k_ = random effect, unobservable, assumed to be NIID (0, σ2 e).

The experiment followed a randomized complete block design, with paddock serving as the blocking factor and parity (primiparous vs. multiparous) as the fixed effect. Data were analyzed using the GLIMMIX procedure of SAS (SAS Institute Inc., Cary, NC, USA, version 9.4), applying the Kenward–Roger method to approximate degrees of freedom for fixed effects. Cow BW, BCS, BW change, BCS change, gestation length and calf BW were tested for fixed effect of parity, whereas blood parameters, milk yield, and milk composition were analyzed for the fixed effects of parity, day of sampling, and parity × day interactions, with sampling day considered with repeated measures over time. The best covariance structure was selected based on the lowest Akaike Information Criterion for each variable. Calf sex was included as a covariate for calf variables. Least square means were separated using the least significant difference. Differences were set at *p* ≤ 0.05 and tendencies were 0.05 < *p* ≤ 0.10.

In the context of this study, parity is not being treated as an isolated factor but rather as a composite indicator that encompasses physiological maturity, developmental stage, lactation experience, and the associated differences in BW and age. Therefore, these variables (i.e., BW and age) were not adjusted as covariate because they are integral to the parity effect of interest.

Data for correlation analysis was partitioned into three physiological periods: late gestation (d−21 to 0), first trimester of lactation (d7 to 42), and second/third trimester of lactation (d63 to 203). Within each period, data from multiparous and primiparous cows were analyzed separately. Pearson correlation coefficients between hormonal variables, blood metabolites, and performance traits were computed using the PROC CORR procedure of SAS (SAS Inst. Inc., Cary, NC). Non-parametric Spearman’s correlations were applied for BCS and all other physiological variables. Correlation results were summarized in correlation matrices, where each cell contained the correlation coefficient (r) and the corresponding *p*-value. The strength of correlations was interpreted as follows: weak (|r| < 0.39), moderate (0.40 ≤ |r| < 0.69), and strong (|r| ≥ 0.70). Only pairwise correlations with |r| ≥ 0.39 were considered biologically relevant, and significance was declared at *p* ≤ 0.05. Correlation matrices were presented by physiological period and parity group to facilitate interpretation of the relationships between metabolic indicators and performance outcomes. Pearson correlation was used for exploratory purposes, and the results should not be considered as definitive associations.

## 3. Results

### 3.1. Cow Performance and Milk Production

Multiparous cows had greater initial, calving, and final BW and BCS (*p* < 0.0001), as well as longer gestation length (*p* = 0.01), heavier calf birth weight (*p* = 0.02) and calf weaning weight (*p* = 0.01) compared with primiparous cows (Table 2). In contrast, primiparous cows tended to gain more BW from days 63 to 203 (*p* = 0.09).

**Figure 1:**
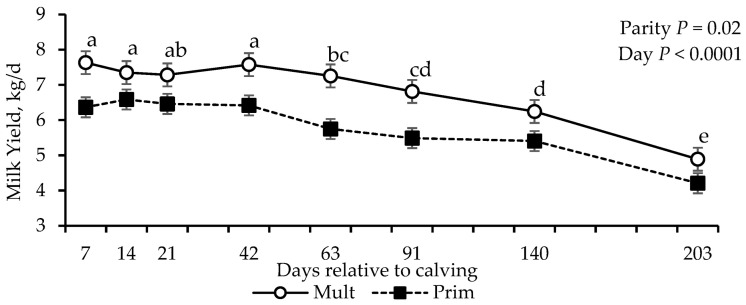
Milk yield (kg) of grazing multiparous (*n* = 17) and primiparous (*n* = 17) cows. ^a–e^ Means without common superscripts differ between days (*p* ≤ 0.05).

Effect of parity (*p* = 0.02) and days (*p* < 0.0001) was detected for milk yield (Table 3), with multiparous cows producing more milk throughout the lactation period. Milk yield remained stable from days 7 to 42, followed by a decline from days 63 to 203, with the lowest yield recorded on day 203 (*p* < 0.01; Figure 1).

Effect parity (*p* < 0.01) and day (*p* < 0.0001) were detected for milk fat and total solids, where multiparous cows showed greater milk fat and total solids content compared to primiparous cows (Table 3). No effect of parity or interaction with days was detected for lactose content (*p* > 0.05; Table 3).

### 3.2. Cow Metabolic and Hormonal Profile

**Figure 2:**
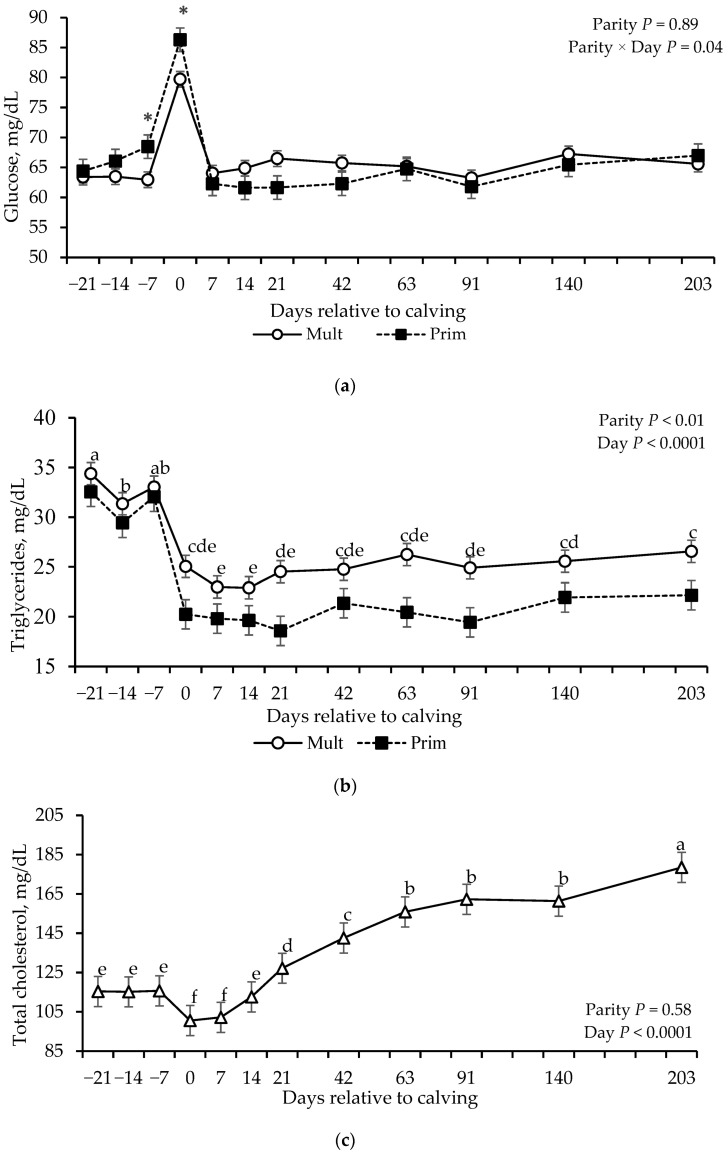

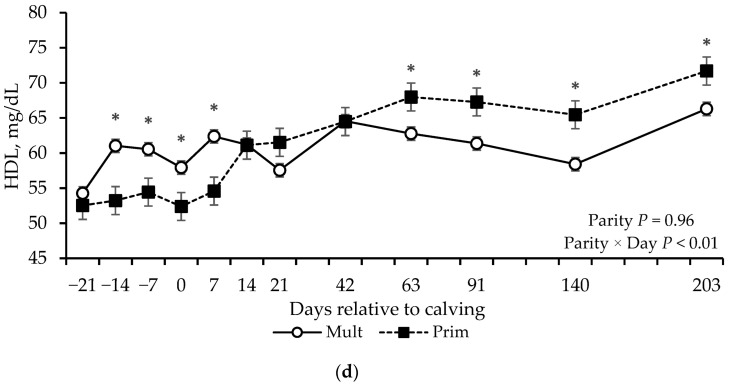
Glucose (**a**), triglycerides (**b**), total cholesterol (**c**) and high-density lipoprotein (HDL, (**d**) concentrations of grazing multiparous (*n* = 17) and primiparous (*n* = 17) cows during late gestation and lactation periods. * shows the difference between parity groups within the day (*p* ≤ 0.05). ^a–f^ Means without common superscripts differ between days (*p* ≤ 0.05).

Effect of parity × day interaction was detected for glucose concentration (*p* = 0.04; Table 4), where primiparous cows showed higher concentrations on days −7 and 0 compared to multiparous cows (Figure 2a). Effect of parity (*p* = 0.01) and days (*p* < 0.0001) were detected for VLDL and triglyceride concentrations (Table 4), with greater concentrations in multiparous cows compared to primiparous cows (Figure 2b). Overall, triglyceride concentrations were higher in the prepartum period compared to the postpartum period, with the lowest values on days 7 and 14.

Effect of day (*p* < 0.0001), but not parity or parity × day was detected for total cholesterol and LDL. Total cholesterol concentrations were lower during prepartum and progressively increased after calving (Figure 2c). Effect of parity × day interaction was found for HDL concentrations (*p* = 0.002; Table 4); multiparous cows had greater concentrations of HDL from d−14 to 7, but lower HDL concentrations from d63 to 203, compared to primiparous cows (Figure 2d).

**Figure 3:**
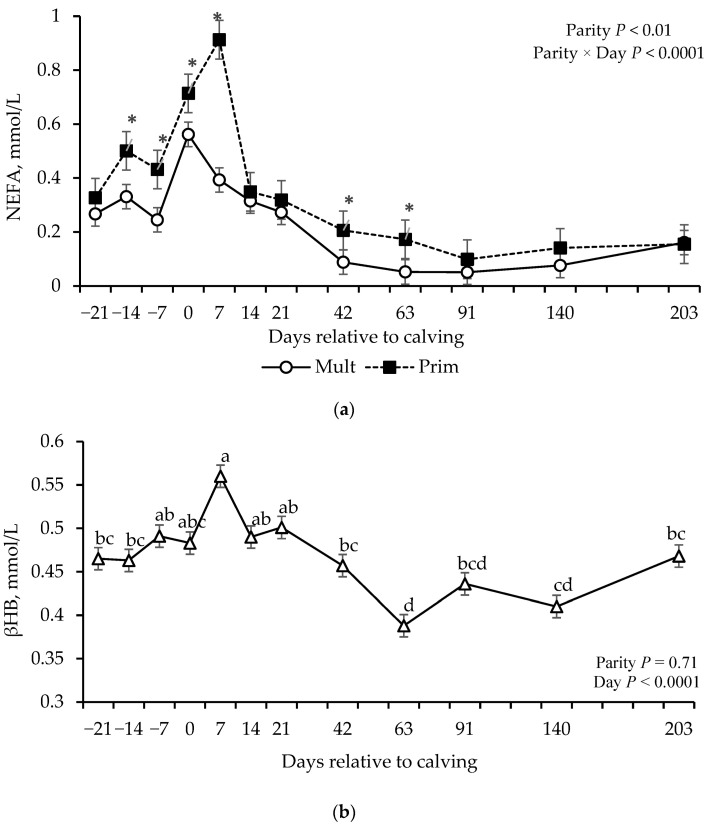
Non-esterified fatty acids (NEFA, (**a**), beta-hydroxybutyrate (βHB), (**b**) concentrations of grazing multiparous (*n* = 17) and primiparous (*n* = 17) cows during late gestation and lactation periods. * shows the difference between parity groups withing day (*p* ≤ 0.05). ^a–d^ Means without common superscripts differ between days (*p* ≤ 0.05).

Effect of parity × day interaction was detected for NEFA concentrations (*p* ≤ 0.01; Table 4), where primiparous cows showed greater NEFA concentrations on days −14, −7, 0, and 7 (Figure 3a). Effect of day (*p* < 0.0001), but not parity or parity × day (*p* > 0.58) was observed for βHB (Table 4). β-hydroxybutyrate concentrations were stable during the prepartum period until calving, increased on day 7, fluctuated between days 14 and 42, decreased on day 63, and fluctuated again from day 91 to 140, followed by an increase on day 204 (Figure 3b).

**Figure 4:**
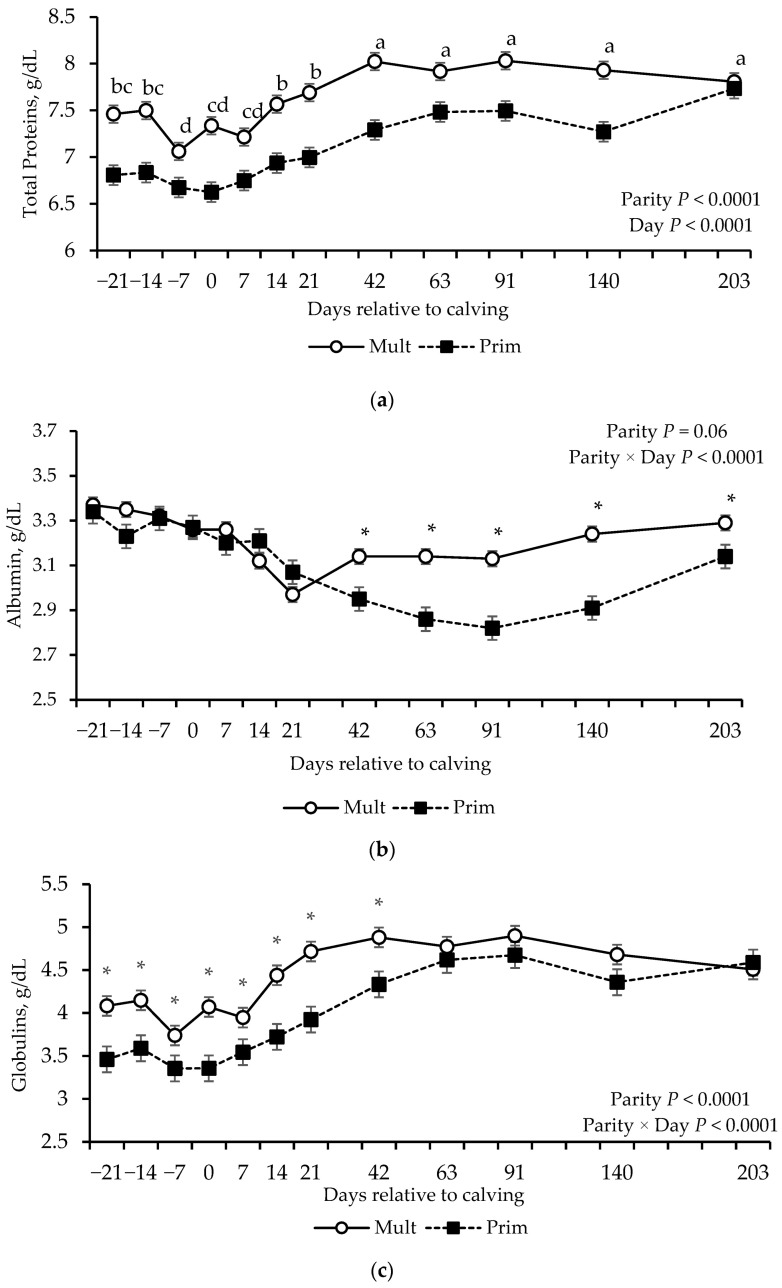

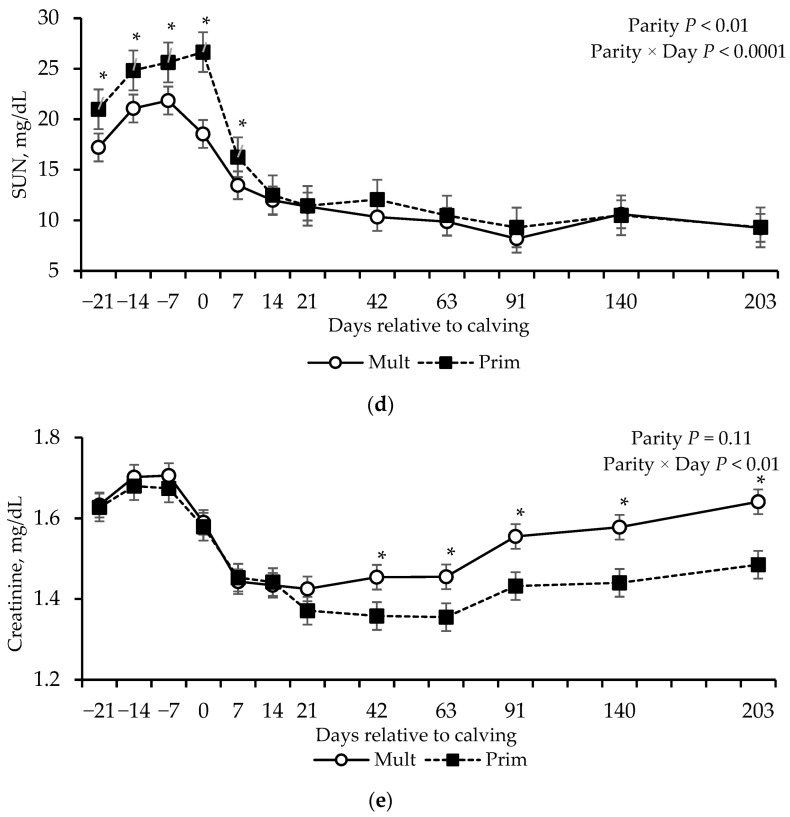
Total proteins (**a**) albumin (**b**) globulins, (**c**) serum urea nitrogen (SUN), (**d**) and creatinine (**e**) concentrations of grazing multiparous (*n* = 17) and primiparous (*n* = 17) cows during late gestation and lactation periods. * shows the difference between parity groups withing day (*p* ≤ 0.05). ^a–d^ Means without common superscripts differ between days (*p* ≤ 0.05).

Effect parity (*p* < 0.01) and day (*p* < 0.0001) were detected for total protein concentrations (Table 4), where multiparous cows showed greater concentration compared to primiparous cows. On average, total protein remained stable from prepartum through day 21 postpartum, followed by an increase at day 42, and remained stable again through day 203 (Figure 4a).

**Figure 5:**
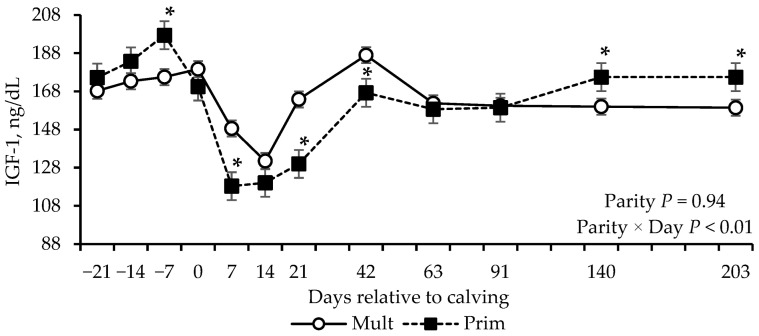
IGF-1 concentrations of grazing multiparous (*n* = 17) and primiparous (*n* = 17) cows during late gestation and lactation periods. * shows the difference between the parity groups withing day (*p* ≤ 0.05).

Effect of parity × day interaction was found for albumin, globulins, SUN, creatinine, and IGF-1 concentrations (*p* ≤ 0.01; Table 4). Multiparous cows had greater albumin concentrations between day 42 and 140 postpartum, and greater globulins concentration from day −21 prepartum through day 42 postpartum compared to primiparous cows (Figure 4b,c). Primiparous cows had greater SUN concentrations during the prepartum period and at calving (Figure 4d). Creatinine concentrations were greater in multiparous cows on days 91, 140, and 203 compared to primiparous cows (Figure 4e). IGF-1 concentrations were greater in multiparous cows on days 7, 21 and 42, but lower on days −7, 140 and 203, compared to primiparous cows (Figure 5).

### 3.3. Pearson Correlation

#### 3.3.1. Multiparous Cows at Late Gestation

**Figure 6:**
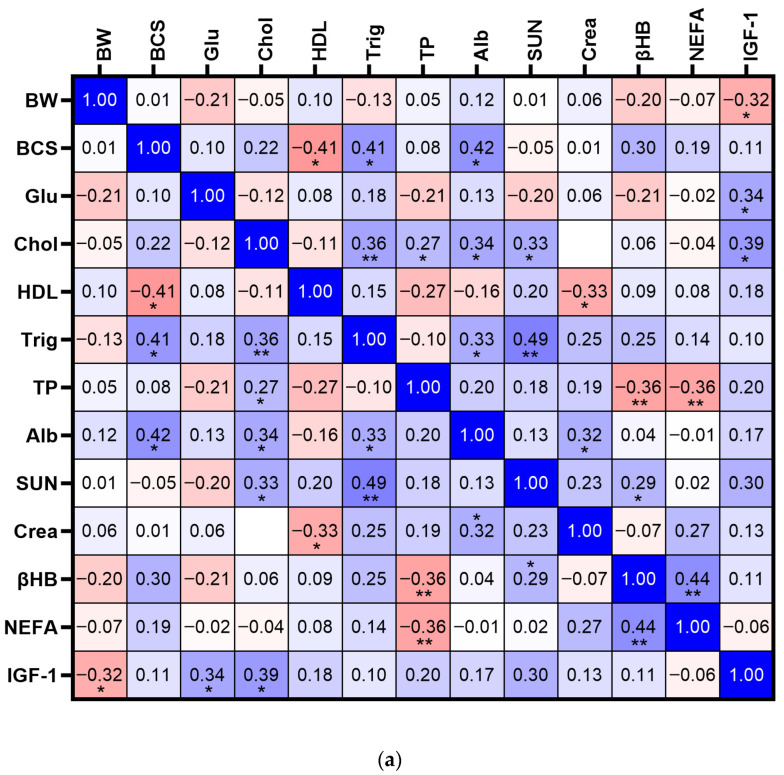

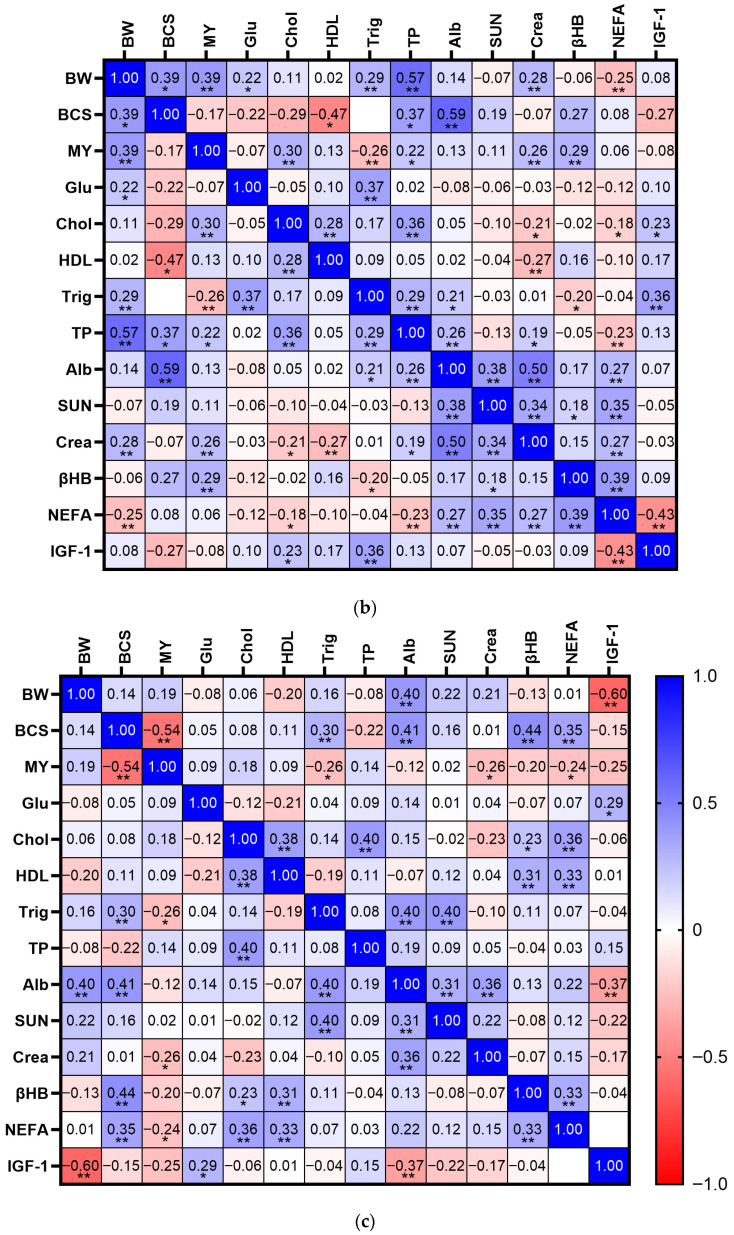
Heatmap of Pearson correlation between hormones, metabolites and performance variables of multiparous beef cows during (**a**) late gestation (days −21 to 0), (**b**) first trimester of lactation (days 7 to 42) and (**c**) second and third trimester of lactation (days 63 to 203). BW: body weight, BCS: body condition score, MY: milk yield, Glu: glucose, Chol: cholesterol, HDL: high-density lipoprotein, Trig: triglycerides, TP: total protein, Alb: albumin, SUN: serum urea nitrogen, Crea: creatinine, βHB: beta-hydroxybutyrate, NEFA: non-esterified fatty acid, IGF-1: insulin-like growth factor 1. * *p* < 0.05, ** *p* < 0.01.

Body weight was negatively correlated with IGF-1 (r = −0.32; *p* < 0.05; Figure 6a). IGF-1 was positively correlated with glucose (r = 0.34; *p* < 0.05). Total cholesterol was positively correlated with triglycerides (r = 0.36; *p* < 0.01), albumins (r = 0.34; *p* < 0.05), SUN (r = 0.33; *p* < 0.05) and IGF-1 (r = 0.39; *p* < 0.05; Figure 6a). BCS was negatively correlated with HDLs (r = −0.41; *p* < 0.05) but positively correlated with triglycerides and albumins (r = 0.42; *p* < 0.05). Non-esterified fatty acids were positively correlated with βHB (r = 0.44; *p* < 0.01) but negatively correlated with total proteins (r = −0.36; *p* < 0.01; Figure 6a).

#### 3.3.2. Multiparous Cows at First Trimester of Lactation

As expected, BW was positively correlated with BCS (r = 0.39; *p* < 0.01; Figure 6b) and moderately correlated with total protein (r = 0.57; *p* < 0.01) and creatinine (r = 0.28; *p* < 0.01). Body condition score also showed a moderate positive correlation with total proteins (r = 0.69; *p* < 0.01) and triglycerides (r = 0.55; *p* < 0.01). Body condition score was negatively associated with HDL (r = −0.59; *p* < 0.05) but positively correlated with total proteins and albumin (r = 0.37; *p* < 0.05). IGF-1 showed a moderate negative correlation with NEFA (r = − 0.43; *p* < 0.01). Non-esterified fatty acids were positively correlated with βHB (r = 0.39; *p* < 0.01). Milk yield showed a weak but significant positive correlation with βHB (r = 0.29; *p* < 0.01), and a negative correlation with triglycerides (r = −0.26; *p* < 0.01; Figure 6b).

#### 3.3.3. Multiparous Cows at Second and Third Trimester of Lactation

Body weight was negatively correlated with IGF-1 (r = −0.60; *p* < 0.01) but showed positive correlations with albumin (r = 0.40; *p* < 0.01; Figure 6c). Body condition score was positively correlated with triglycerides (r = 0.30; *p* < 0.01), albumin (r = 0.41; *p* < 0.01), and NEFA (r = 0.35; *p* < 0.01), and negatively correlated with MY (r = −0.54; *p* < 0.01). Milk yield was moderately and negatively associated with BCS (r = −0.54; *p* < 0.01) and negatively correlated with creatinine (r = −0.26; *p* < 0.05), triglycerides (r = −0.26; *p* < 0.05), and NEFA (r = −0.24; *p* < 0.05; Figure 6c).

#### 3.3.4. Primiparous Cows at Late Gestation

**Figure 7:**
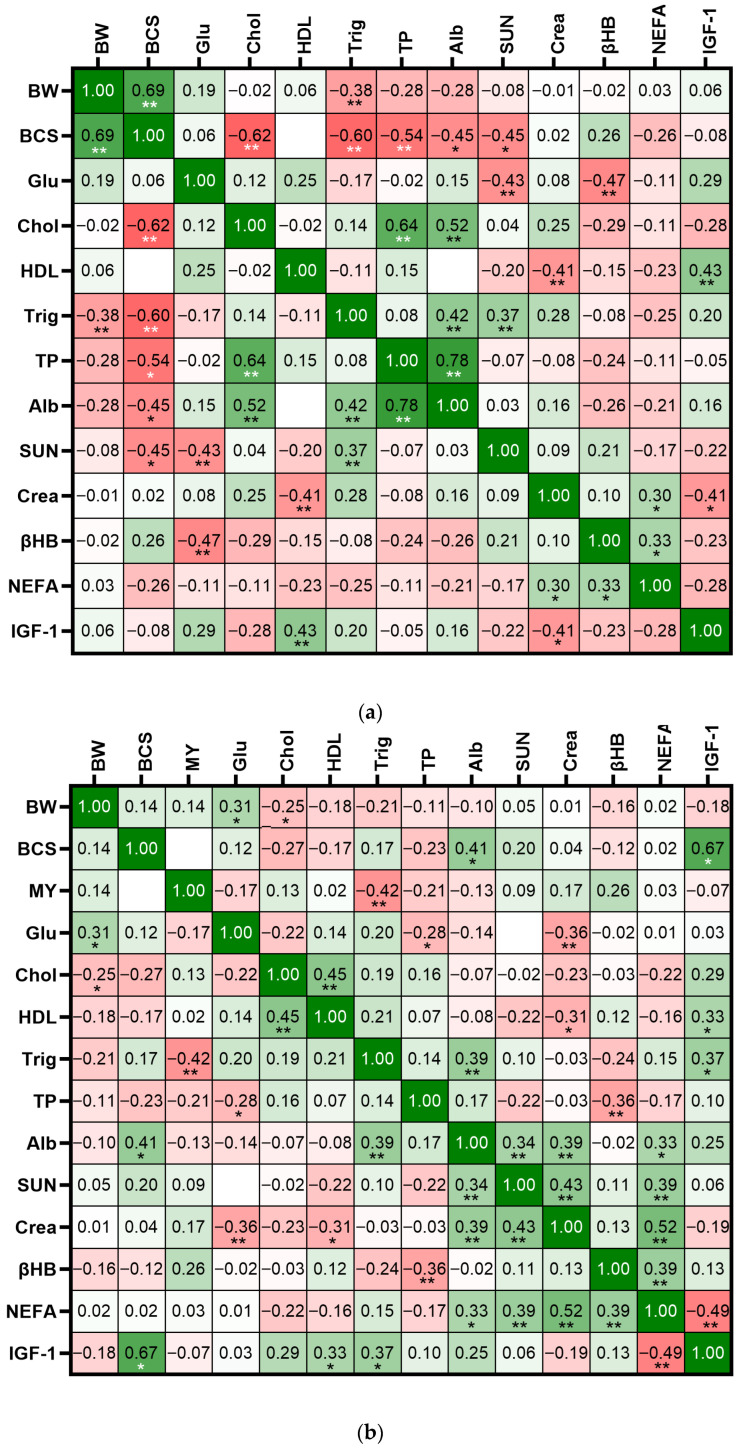

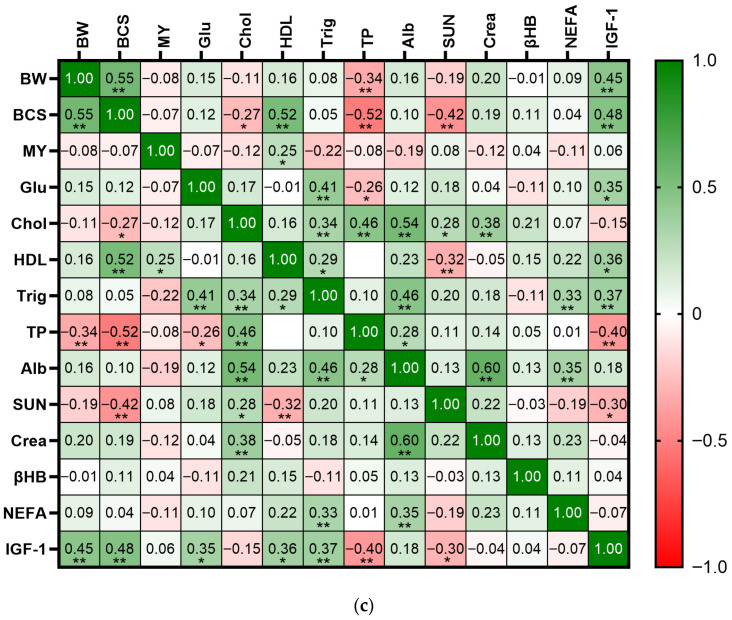
Heatmap of Pearson correlation between hormones, metabolites and performance variables of primiparous beef cows during (**a**) late gestation (days −21 to 0), (**b**) first trimester of lactation (days 7 to 42) and (**c**) second and third trimester of lactation (days 63 to 203). BW: body weight, BCS: body condition score, MY: milk yield, Glu: glucose, Chol: cholesterol, HDL: high-density lipoprotein, Trig: triglycerides, TP: total protein, Alb: albumin, SUN: serum urea nitrogen, Crea: creatinine, βHB: beta-hydroxybutyrate, NEFA: non-esterified fatty acid, IGF-1: insulin-like growth factor 1. * *p* < 0.05, ** *p* < 0.01.

Body weight was strongly and positively correlated with BCS (r = 0.69, *p* < 0.01) and negatively correlated with triglycerides (r = −0.38, *p* < 0.01). Glucose was negatively associated with SUN (r = −0.43, *p* < 0.01) and βHB (r = −0.47, *p* < 0.01; Figure 7a). Body condition score was negatively correlated with HDL (r = −0.62), triglycerides (r = −0.60), total proteins (r = −0.54), albumin (r = −0.45) and SUN (r = −0.45, *p* < 0.05). Moderate positive correlations were observed between cholesterol and total protein (r = 0.64, *p* < 0.01) and albumin (r = 0.52, *p* < 0.01). Likewise, total protein and albumin were highly correlated (r = 0.78, *p* < 0.01), as expected, and were also positively related to triglycerides (r = 0.42, *p* < 0.01). Creatinine was negatively correlated with IGF-1 (r = −0.41, *p* < 0.01). IGF-1 was positively associated with HDL (r = 0.43, *p* < 0.01; Figure 7a).

#### 3.3.5. Primiparous Cows at First Trimester of Lactation

Body weight was positively correlated with glucose (r = 0.31, *p* < 0.05) but negatively correlated with cholesterol (r = −0.25, *p* < 0.05). Body condition score was moderately and positively correlated with albumin (r = 0.41, *p* < 0.01) and IGF-1 (r = 0.67, *p* < 0.05; Figure 7b). Milk yield was negatively correlated with triglycerides (r = −0.42, *p* < 0.01). A positive relationship was observed between cholesterol and HDLs (r = 0.45, *p* < 0.01) and between triglycerides and albumin (r = 0.39, *p* < 0.01). Additionally, NEFA was moderately correlated with creatinine (r = 0.52, *p* < 0.01), SUN (r = 0.39, *p* < 0.01) and βHB (r = 0.39, *p* < 0.01). IGF-1 showed a moderate and positive correlation with BCS (r = 0.67, *p* < 0.05) and negative correlation with NEFA (r = −0.49, *p* < 0.01; Figure 7b).

#### 3.3.6. Primiparous Cows at Second and Third Trimester of Lactation

Body weight was positively correlated with BCS (r = 0.55, *p* < 0.01) and IGF-1 (r = 0.45, *p* < 0.01; Figure 7c). However, BW was negatively correlated with total protein (r = −0.34, *p* < 0.01). Body condition score was also positively correlated with IGF-1 (r = 0.48, *p* < 0.01) and HDL (r = 0.52, *p* < 0.01) and negatively associated with SUN (r = −0.42, *p* < 0.01), total protein (r = −0.52, *p* < 0.01), and cholesterol (r = −0.27, *p* < 0.05). IGF-1 showed multiple significant correlations, being positively associated with glucose (r = 0.35, *p* < 0.05), HDL (r = 0.36, *p* < 0.05), and triglycerides (r = 0.37, *p* < 0.01). Conversely, it was negatively associated with total protein (r = −0.40, *p* < 0.01) and SUN (r = −0.30, *p* < 0.05; Figure 7c).

Triglycerides had positive correlations with albumin (r = 0.46, *p* < 0.01), glucose (r = 0.41, *p* < 0.01) and cholesterol (r = 0.34, *p* < 0.01). Cholesterol was positively associated with total protein (r = 0.46, *p* < 0.01), albumin (r = 0.54, *p* < 0.01), and SUN (r = 0.28, *p* < 0.05; Figure 7c).

## 4. Discussion

This study aimed to characterize metabolic and physiological differences between primiparous and multiparous beef cows during late gestation and lactation and to explore how these differences relate to productivity. Primiparous cows showed higher concentrations of glucose one week before parturition and at calving, compared to multiparous cows. This is likely related to more excitable temperaments, and stress and fear experienced during calving relative to older cows [15]. Elevated stress hormones such as cortisol and epinephrine stimulate glycogenolysis, thereby increasing circulating glucose. In addition, a prepartum rise in blood glucose concentration is a well-recognized indicator of approaching parturition in cattle [16], which aligns with the shorter gestation length observed in primiparous cows in this study. Thus, these animals were physiologically closer to calving than multiparous cows at the time of sampling. Similar findings of greater glucose concentrations in primiparous cows around calving have been reported in other studies [4,16,17]. Ferreira et al. [4] observed greater glucose concentrations for primiparous beef cows at calving in similar conditions, whereas Janovick [16] observed greater glucose concentrations for primiparous cows at calving and during early postpartum for dairy cows.

Primiparous cows had elevated NEFA concentrations around calving and early lactation, reflecting a greater negative energy balance due to the simultaneous demands of growth and milk production. This is in agreement with others [10,11,17] who reported higher NEFA concentrations in primiparous cows compared with multiparous dairy cows during early lactation. In contrast, other studies in dairy cows observed greater NEFA concentrations in multiparous cows during lactation [5,18], which was attributed to the higher milk yields of this category. Differences between studies could be related to production system management, breed, nutrition, and sampling time. In the present study, βHB did not differ between parities; however, NEFA and βHB were positively correlated during early lactation in both groups. Because both metabolites are strong indicators of catabolism in dairy cattle, a positive correlation is expected. Nonetheless, NEFA generally appears to be a more sensitive indicator of fat mobilization in beef cows [19]. Although NEFA and glucose concentrations differed between parity groups, the magnitude of differences was greater for indicators of protein status (e.g., albumin, total protein, SUN and IGF-1).

Multiparous cows typically have higher concentrations of albumin and globulins compared to primiparous cows, which also leads to differences in total proteins in the blood [4,5]. The difference in albumin concentration is likely due to the deviation of amino acids from albumin synthesis to other body tissues as a homeorhetic mechanism since these categories require nutrients for fetus development, lactation, and continued growth. On the other hand, globulins are not exactly great indicators of the protein status of an animal, being much more related to inflammatory responses and immunity [20]. Greater concentrations of globulin for multiparous cows compared to primiparous cows have been reported for dairy [21,22,23] and beef cows [4] and are related to a more mature immunological memory of older animals.

On the other hand, primiparous cows exhibited higher SUN concentrations during late gestation and early postpartum, which could reflect increased mobilization of muscle protein to meet energy demands rather than dietary differences, as all animals grazed in the same pastures [4]. Conversely, other studies observed greater SUN concentrations for multiparous cows compared to primiparous cows for dairy [5,11] and beef cows [24].

Both parity groups showed positive associations between cholesterol and protein-related markers (albumin and total protein), indicating that lipoprotein metabolism and protein status are tightly linked in late gestation regardless of parity. Similarly to blood proteins, cholesterol concentrations in the blood can be related to dry matter intake and nutrient uptake, which also explains lower value for these metabolites around calving [8]. The progressive increase in cholesterol levels throughout postpartum days agrees with other studies [8,25].

In early lactation, similarities emerged between groups in the negative correlation between MY and triglycerides, supporting the idea that greater milk production may drive lipid mobilization or depletion of circulating lipids in both parity groups. However, several important distinctions were observed. In multiparous cows, BW and BCS showed very strong positive correlations and were positively associated with creatinine and total protein.

During early lactation, IGF-1 was negatively correlated with NEFA for both primiparous and multiparous cows [5], showing the antagonism of these two variables. Low IGF-1 and high NEFA concentrations for both dairy and beef are related to poor nutritional status and negative energy balance early lactation.

The concentration of IGF-1 between cows of parity groups has been shown to be inconsistent where studies have shown great IGF-1 concentrations for multiparous cows [5], compared to primiparous cows, where others have observed the opposite [10]. In this study, interestingly, IGF-1 was lower for primiparous cows during the first weeks of lactation, confirming their poorer nutritional and metabolic status compared with multiparous cows [4]. However, as lactation progressed and milk yield declined, IGF-1 concentrations in primiparous cows increased, surpassing those of multiparous cows by the late lactation period. This rebound coincided with greater BW gains observed in primiparous cows from day 63 to 203. Correlation analysis further supports these patterns: in multiparous cows, the correlation between BW and IGF-1 during the last trimester of lactation was negative, and MY was negatively associated with multiple markers, including BCS, creatinine, and NEFAs, reinforcing the energy-demanding nature of sustained lactation in mature cows. The strong negative correlation between MY and BCS emphasizes the physiological cost of milk production on body reserves in this group. Conversely, in primiparous cows, BW and BCS were positively correlated with IGF-1, and BCS was negatively associated with SUN and cholesterol. These patterns suggest a more anabolic profile in the later lactation period for primiparous cows, potentially reflecting a recovery in growth or improved adaptation after the metabolic strain of early lactation. Additionally, IGF-1 maintained positive correlations with energy-related markers such as glucose and triglycerides, reinforcing its role as an indicator of nutritional status [26].

In this study, calves born to primiparous dams had 13% lower birth weight and 8% lower weaning weights than calves born to multiparous dams, despite having the same sire. This percentage for birth weights is comparable to the 10.5% and 10.5% reductions observed by Duncan et al. [24] and Bellows et al. [27]. Although the competition of nutrient partitioning between maternal growth and pregnancy may result in decreased fetal growth for calves born to heifers [28], the differences in birth weight could also be aggravated by differences in gestation length, being shorter for primiparous cows. Gestation length differences due to parity have been variable in beef cattle [24,29,30].

These differences observed between primiparous cows and multiparous cows may reflect similar fetal programming responses triggered by nutritional or stress-related challenges during gestation. For example, studies have shown that calves born from dams on low nutrition during late gestation had birth weights that were approximately 8% lower and weaning weights about 10% lighter than those from dams with adequate nutrition [31,32]. These parallels suggest that parity-related differences in our study are within the range of effects typically attributed to maternal nutritional programming.

Differences in energy and protein metabolism led to lower milk production in primiparous cows, as the differing metabolic traits may limit the partitioning of nutrients into milk. Consistent with previous reports in both dairy [33,34] and beef cows [12,35], multiparous cows produced more milk across lactation compared with primiparous cows. Multiparous cows also showed greater milk fat and total solids content, reinforcing the advantage of maturity and established mammary development in supporting milk synthesis. Lower milk yield and poorer nutritional composition for primiparous cows, as a consequence of nutrient partitioning toward maternal growth at the expense of lactation [36], contributed to reduced calf weaning weight compared to multiparous cows.

## 5. Conclusions

Overall, multiparous cows showed a less catabolic metabolic profile during the periparturient period to lactation. Primiparous cows experienced a more intense mobilization of body reserves during the early lactation period, but a more positive nutritional status towards late lactation was likely associated with the reestablishment of continued growth. Primiparous cows had higher NEFA, SUN and glucose concentrations around calving, and lower concentrations of IGF-1, total proteins, albumin and globulins, compared to multiparous cows. The combination of a less-balanced metabolic profile during late gestation and early lactation, and lower milk yield in primiparous cows was associated with reduced calf birth and weaning weights compared with multiparous cows.

Correlation analysis revealed distinct metabolic and physiological interrelationships across gestational and lactational stages in multiparous and primiparous cows. In summary, correlation analysis highlighted IGF-1 as a central metabolic marker, showing negative associations with NEFA and positive correlations with glucose and triglycerides, particularly during early lactation. These patterns reflect the antagonism between anabolic and catabolic states and highlight IGF-1’s sensitivity to energy balance. Milk yield was negatively correlated with BCS, NEFA, and triglycerides, especially in multiparous cows. In contrast, primiparous cows showed positive correlations between IGF-1, BCS, and HDL during late lactation, suggesting a shift toward recovery and growth. These findings highlight the importance of targeted nutritional strategies for primiparous cows to improve productivity in pasture-based beef systems. Improving metabolic balance in primiparous cows could enhance calf performance and overall herd productivity in pasture-based beef systems.

## Figures and Tables

**Table 1:** Average chemical composition and forage mass of *Urochloa decumbens* pastures according to season, and chemical composition of energy-protein supplements.

Items ^1^	Supplement	*Urochloa decumbens*				SD
		Dry	Dry–Rainy	Rainy	Rainy–Dry	
DM, g/kg	870.9	338.4	408.2	370.2	356.0	19.7
CP, g/kg	32.1	67.1	83.1	95.3	70.7	4.03
NDF, g/kg	146.6	681.6	699.1	671.3	711.4	23.4
iNDF, g/kg	-	282.9	288.4	230.4	262.4	12.6
NDIN, g/kg	-	25.5	23.0	23.8	22.5	0.92
Forage mass (kg DM/ha)	-	2600	3500	3200	3100	490.3

^1^ DM, dry matter; CP, crude protein; NDF, neutral detergent fiber; iNDF, indigestible neutral detergent fiber; NDIN, neutral detergent insoluble nitrogen. SD, standard deviation (largest observed SD among seasons for each item).

**Table 2:** Body weight, body condition score, and calf growth performance of grazing multiparous (*n* = 17) and primiparous (*n* = 17) of Nellore cows.

Items ^1^	Parity Group			*p*-Value
	Multiparous	Primiparous	SEM	Parity
Initial body weight, kg	597	407	9.40	<0.0001
Change d−63 to d−7	5.2	3.1	1.94	0.61
Calving BW, kg	543	366	10.1	<0.0001
Change d7 to d63	−1.5	−7	1.64	0.29
Change d63 to d203	17.6	27.5	3.21	0.09
Final body weight, kg	577	406	10.62	<0.0001
Initial BCS (1−9)	6.6	5.5	0.11	<0.0001
Change d−63 to d0	−0.4	−0.3	0.08	0.82
Calving BCS (1–9)	6.2	5.2	0.09	<0.0001
Change d0 to d63	−0.6	−0.4	0.09	0.75
Change d63 to d203	1.1	0.6	0.10	0.18
Final BCS (1–9), kg	6.8	5.5	0.08	<0.0001
Gestation length, days	286	281	1.08	0.01
Calf birth weight, kg	35.0	30.3	1.29	0.02
Calf weaning weight, kg	229	212	5.91	0.01

^1^ Considering calving day as day 0, cow full BW was collected on days −63, −14, −21, −7, 0, 7, 14, 7, 21, 63, 91, 140 and 203, and BCS was collected on days −63, −21, 0, 21, 63, 91, 140 and 203. Day −63—initial BW and BCS, day 63—beginning of breeding season and day 204—final BW and BCS (weaning). Gestation length was calculated based on the insemination day and calving day (d0). Calf sex (*p* ≥ 0.17) was included as a covariate for calf variables.

**Table 3:** Milk yield and composition of grazing multiparous (*n* = 17) and primiparous (*n* = 17) Nellore cows.

Items	Parity Group			*p*-Value		
	Multiparous	Primiparous	SEM	Parity	Day	Parity × Day
Milk yield, kg/d	6.88	5.83	0.350	0.02	<0.0001	0.31
Fat, g/100 g	4.76	4.23	0.098	<0.001	<0.0001	0.32
Protein, g/100 g	3.09	3.10	0.112	0.90	0.02	0.29
Lactose, g/100 g	4.70	4.67	0.040	0.66	0.12	0.35
Total solids, g/100 g	13.28	12.66	0.132	0.01	<0.0001	0.35

Milk yield was measured in the morning and afternoon on days 8, 15, 22, 43, 64, 92, 141 and 204, as described by Ferreira et al. [12]. Total milk yield was calculated as the sum of both morning and afternoon milking. Additionally, 30 mL of milk from each cow was sampled for milk composition analyses.

**Table 4:** Metabolic and hormonal profile during late gestation and lactation of grazing multiparous (*n* = 17) and primiparous (*n* = 17) Nellore cows.

Items ^1^	Parity Group			*p*-Value ^2^		
	Multiparous	Primiparous	SEM	Parity	Day	Parity × Day
Glucose, mg/dL	65.99	66.09	0.526	0.89	<0.0001	0.04
Triglycerides, mg/dL	26.86	23.14	0.780	<0.01	<0.0001	0.41
Total cholesterol, mg/dL	133.72	131.22	3.193	0.58	<0.0001	0.77
VLDL, mg/dL	5.37	4.63	0.156	<0.01	<0.0001	0.41
LDL, mg/dL	66.95	64.90	3.424	0.67	<0.0001	0.64
HDL, mg/dL	60.69	60.56	1.845	0.96	<0.0001	<0.01
Total proteins, g/dL	7.63	7.08	0.085	<0.0001	<0.0001	0.12
Albumin, g/dL	3.22	3.11	0.038	0.06	<0.0001	<0.0001
Globulins, g/dL	4.41	3.96	0.081	0.001	<0.0001	<0.01
SUN, mg/gL	13.65	15.83	0.533	0.01	<0.0001	<0.0001
Creatinine, mg/dL	1.55	1.49	0.026	0.11	<0.0001	<0.01
NEFA, nmol/L	0.24	0.36	0.024	<0.01	<0.0001	<0.0001
βHB, nmol/L	0.47	0.46	0.015	0.72	<0.0001	0.61
IGF-1, ng/mL	162.96	161.91	10.565	0.94	<0.0001	<0.01

^1^ VLDL—very-low-density lipoprotein, LDL—low-density lipoprotein, HDL—high-density lipoprotein, SUN—serum urea nitrogen, NEFA—non-esterified fatty acids; βHB—beta-hydroxybutyrate; IGF-1—insulin-like growth factor 1. ^2^ Interaction between parity × day (*p* ≤ 0.04) was detected for glucose, HDLs, albumins, globulins, SUN, NEFAs and IGF-1.

## Data Availability

The original contributions presented in this study are included in the article. Further inquiries can be directed to the corresponding author.

## Abbreviations

The following abbreviations are used in this manuscript:

BCS	Body condition score
NEFA	Non-esterified fatty acids
IGF-1	Insulin-like growth factor 1
BW	Body weight
HDL	High-density lipoprotein
LDL	Low-density lipoprotein
βHB	β-hydroxybutyrate
CP	Crude protein
DM	Dry matter
NDF	Neutral detergent fiber
iNDF	Indigestible neutral detergent fiber
NDIN	Neutral detergent insoluble nitrogen
SUN	Serum urea nitrogen
MY	Milk Yield
Glu	Glucose
Trig	Triglycerides
TP	Total protein
Alb	Albumin
Crea	Creatinine
Chol	Cholesterol
